# Post‐menopausal oestrogen deficiency induces osteoblast apoptosis via regulating HOTAIR/miRNA‐138 signalling and suppressing TIMP1 expression

**DOI:** 10.1111/jcmm.16216

**Published:** 2021-03-17

**Authors:** Shao‐Yong Xu, Peng Shi, Rui‐Ming Zhou

**Affiliations:** ^1^ Division of Orthopedics and Traumatology Department of Orthopedics Nanfang Hospital Southern Medical University Guangzhou China; ^2^ Department of Spine Orthopedics Liuzhou Traditional Chinese Medical Hospital Liuzhou China; ^3^ Department of Orthopedics The Second People Hospital of NanSha Guangzhou China

**Keywords:** HOTAIR, menopause, miR‐138, oestrogen, osteoporosis, TIMP1

## Abstract

In this study, we aimed to explore the molecular mechanisms underlying the development of osteoporosis in post‐menopausal females. Real‐time PCR was conducted to measure the expression of potential lncRNAs involved in the osteoporosis of post‐menopausal females. In addition, Western blot and IHC assays were used to study the possible correlation among HOTAIR, miR‐138 and TIMP1, while a computational analysis was carried out to predict the ‘seed sequence’ responsible for the binding between miR‐138 and HOTAIR/TIMP1. Furthermore, luciferase reporter assays were conducted to validate the negative regulatory relationship between miR‐138 and TIMP1/HOTAIR. To evaluate the effect of oestrogen on the function of HOATIR and its downstream effectors, luciferase activity was measured in cells cotransfected with different vectors or treated with different doses of oestrogen. The results of the luciferase assay were further validated by real‐time PCR, Western blot, MTT assay and flow cytometry. Among the candidate lncRNAs, HOTAIR was the only lncRNA down‐regulated in post‐menopausal females. HOTAIR bound to miR‐138 and negatively regulated its expression. Meanwhile, miR‐138 could also bind to TIMP1 mRNA and reduce its expression. Furthermore, a dose‐dependent up‐regulation of HOTAIR was observed in cells treated with oestrogen, and the elevated HOTAIR increased the level of TIMP1 by targeting miR‐138. In addition, oestrogen promoted cell viability and suppressed cell apoptosis, and effects of oestrogen were blocked by the silencing of HOTAIR. Therefore, it can be concluded that oestrogen deficiency could induce the apoptosis of osteoblasts and lead to osteoporosis in post‐menopausal females via modulation of the HOTAIR/miR‐138/TIMP1 signalling axis.

## 1 INTRODUCTION

As a disease caused by the loss of bone mineral content and featured by bone strength reduction, osteoporosis affects the life quality of millions of people worldwide, especially those suffering from pathological fractures.^1^, ^2^ Bone re‐modelling tends to maintain a balance between the formation of new bones via osteoblast activation and the resorption of existing bones. Increasing evidence has demonstrated that the apoptosis of osteoblasts is a major mechanism underlying the pathogenesis of osteoporosis.^3^ Therefore, the induction of osteoclast apoptosis and the prevention of osteoblast apoptosis may be used as the potential strategy in the treatment of osteoporosis.^3^

Long non‐coding RNAs (lncRNAs) are RNA transcripts of longer than 200 nucleotides that do not encode proteins.^4^ Although lncRNAs were once deemed as transcriptional ‘noises’, they have been recently shown to play critical roles in many biological processes, such as cell proliferation, transcriptional regulation and tumorigenesis.^5^, ^6^, ^7^ It has been reported that many lncRNAs can ‘sponge’ the activity of microRNAs (miRNAs) by sharing a common miRNA response element (MRE) and inhibiting the expression of their ‘target’ miRNAs.^8^, ^9^ Furthermore, lncRNAs such as HOTAIR have been implicated in osteoblast differentiation.^10^ In addition, the effect of SMAD7 on osteoblast differentiation was also investigated by down‐regulating its expression using miR‐17‐5p. The results of many previous studies collectively suggested that HOTAIR participates in the control of osteoblast differentiation by regulating the expression of miR‐17‐5p and its target gene, SMAD7. So far, several microRNAs (miRNAs), including miR‐214, miR‐148a, miR‐10 and miR‐9, have been implicated in the development of osteoporosis.^11^, ^12^, ^13^ For example, miR‐138 was demonstrated to suppress osteoblast differentiation in mesenchymal stem cells (MSCs), indicating that this miRNA plays a critical role in the progression of osteoporosis. It has also been shown that miR‐138 acts as a negative regulator during the differentiation of human MSCs into osteoblasts, while the inhibition of miR‐138 expression can promote such differentiation.

Previous studies have demonstrated that the overexpression of HOTAIR suppressed the expression of miR‐138 during lipopolysaccharide (LPS)‐stimulation in chondrocytes and rats with rheumatoid arthritis (RA). In addition, the regulatory relationship between miR‐138 and HOTAIR has been further clarified by luciferase assays. In summary, the above results suggested that miR‐138 acts as a target gene of HOTAIR.^14^

Past studies have shown that the decline in the level of ovarian oestrogen during menopause could lead to a rapid loss of trabecular micro‐architecture, thus increasing the rate of bone resorption and the severity of cortical porosity, which in turn increase the risk of osteoporosis and fracture.^15^ Oestrogen deficiency can affect skeletal homeostasis by inducing bone resorption, and the severity of oestrogen deficiency becomes greater over time.^16^ Interestingly, oestrogen deficiency was shown to alter the local distribution of mineral density, thus modifying the mechanical properties of the bones.^17^, ^18^, ^19^, ^20^, ^21^ Furthermore, it has been demonstrated that the onset of oestrogen deficiency can lead to bone loss in the mandible.^22^, ^23^, ^24^

LncRNAs MALAT1 and HOTAIR not only serve as transcriptional targets of miRNAs but are also involved in the regulation of hormone‐sensitive genes, such as PSA, hTERT and pS2, that are targeted by oestrogen in prostate cancer cells.^25^ Previous studies have shown the correlation between the significant reduction in the expression of tissue inhibitor of metalloproteinase‐1 (TIMP1) and increased apoptosis of osteoblasts under endoplasmic reticulum (ER) stress. In summary, TIMP1 exerts a critical effect on osteoblast apoptosis and the development of osteoporosis.^26^ In addition, in prostate cancer cells, oestrogen can up‐regulate the expression of HOTAIR, a competing endogenous RNA for miR‐138.^25^, ^27^ By utilizing an online miRNA database, we identified TIMP1 as a possible target of miR‐138. TIMP1 has been implicated in the development of osteoporosis by regulating the apoptosis of osteoblasts.^27^ In this study, we investigated the effect of oestrogen on the expression of HOTAIR and the activity of its downstream signalling pathways.

## 2 MATERIALS AND METHODS

### 2.1 Human subjects and sample collection

A total of 34 female patients suffering from osteoporosis (with BMD 2.5 standard deviations below the average value according to the WHO criteria) were enrolled in this study. The demographic parameters, risk factors and BMD of all recruited patients were presented in Table 1. Among these participants, 18 patients were post‐menopausal females and were allocated to the Post‐Menopausal Group (group 1). The remaining 16 females were assigned to the Pre‐Menopausal Group (group 2). In addition, bone tissue samples were collected from tibia of each subject during surgical treatment of bone fracture. The tissue samples were lysed and boiled before being loaded on PAGE gel for the subsequent Western blot analysis. This study was approved by the Ethics Committee of our institution and all subjects have signed the informed consent form.

**TABLE 1:** Summary of demographic parameters, risk factors and BMD in post‐ and pre‐menopausal females

Parameter	Post‐menopausal females (N = 18)	Pre‐menopausal females (N = 16)	*P* value
Age (y)	65.8 ± 7.3	45.2 ± 2.3	<.01
Height (cm)	154.8 ± 6.4	155.1 ± 7.1	.4218
Weight (kg)	59.6 ± 7.7	58.6 ± 10.4	.9215
Smoking			
No	3 (16.67)	3 (18.75)	.8744
Current or former	15 (83.33)	13 (81.25)	
Drinking			
No	5 (27.8)	4 (25.0)	.8537
Current or former	13 (72.2)	12 (75.0)	
BMD (g/cm^2^)			
L_1_‐L_4_ Vertebrae	0.90 ± 0.12	0.95 ± 0.11	<.001
Femoral	0.60 ± 0.08	0.67 ± 0.03	<.001
Total hip	0.60 ± 0.07	0.65 ± 0.08	<.001
Trochanter	0.60 ± 0.10	0.65 ± 0.07	<.001

### 2.2 RNA isolation and real‐time PCR

Real‐time PCR was used to compare the expression of multiple lncRNAs, that is HOTAIR, HULC, UCA1, NALAT1 and H19, as well as the expression of miR‐138 and TIMP1 mRNA in the two experimental groups. In brief, the collected bone tissue samples were lysed by TRIZOL (Invitrogen) to isolate total RNA. The concentration and purity of RNA were tested by ultraviolet spectroscopy. Subsequently, 1 µg of purified RNA was collected from each sample and reversely transcribed into cDNA using a reaction system containing 1 μL of oligo (dT) primer, 4 μL of reverse transcription buffer (5×), 2 μL of 10 mmol/L dNTP, 1 μL of RNAase, 1 μL of AMV reverse transcriptase and 11 μL of distilled water. The reverse transcription was carried out at 42°C for 60 minutes, 70°C for 10 minutes and 4°C for 1 hour. Subsequently, real‐time PCR amplification was done on a PE9600 PCR amplification instrument (Perkin‐Elmer). The reaction system of real‐time PCR contained 1.0 μL of buffer (10×), 0.8 μL of 25 mmol/L MgCl_2_, 0.2 μL of 10 mmol/L dNTPs, 0.2 μL of 5 U/μL Taq enzyme, 0.2 μL each of forward primer and reverse primer, 0.2 μL of 0.1 µg/μL cDNA template and 7.6 μL of ddH_2_O. The conditions of real‐time PCR were as follows: pre‐denaturation at 94°C for 2 minutes, denaturation at 94°C for 30 seconds, annealing for 30 seconds and extension at 72°C for 30 seconds.

### 2.3 Cell culture and transfection

HFOB and MG63 cells were cultured in a DMEM medium containing 10% foetal bovine serum (charcoal‐stripped medium was used for oestrogen treatment assay and regular medium for others). When the cells reached approximately 80% confluence, they were adjusted to a concentration of 2 × 10^6^/mL and seeded into 96‐well plates for transfection. The transfection was performed using Lipofectamine 2000 (Invitrogen) according to the instructions of the manufacturer. The cells were harvested at 48 hours post‐transfection to evaluate their expression of target genes. In addition, to investigate the effect of oestrogen on the expression of HOTAIR and TIMP1, HFOB and MG63 cells were respectively grouped into a NC group (negative control), a 5 nmol/L E2 group ( cells treated with 5 nmol/L of oestrogen), a 10 nmol/L E2 group (cells treated with 10 nmol/L of oestrogen), a 10 nmol/L E2 + NC siRNA group (cells treated with 10 nmol/L oestrogen and transfected with negative control siRNAs) and a 10 nmol/L E2 + HOTAIR siRNA group (cells treated with 10 nmol/L oestrogen and transfected with HOTAIR siRNAs). The cells were treated with oestrogen for 24 hours before the expression of HOTAIR, miR‐138 and TIMP1 in the cells was measured.

### 2.4 Cell proliferation assay

Transfected HFOB and MG63 cells were collected and adjusted to a density of 20,000 cells/ml. Subsequently, the cells were seeded into a 96‐well plate and cultured at 37℃ and 5% CO_2_. Each treatment group was assayed in triplicate. In the next step, 20 μL of MTT reagent (5 mg/mL, Sigma‐Aldrich) were added into each well and incubated in the dark for 4 hours at 37°C. Finally, after the addition of 150 μL of DMSO followed by 10 minutes of incubation on a shaker to dissolve the precipitates, the absorbance value in each well was measured on a TFC instrument (Thermo Fisher Scientific) at a wavelength of 490 nm. A growth curve of the cells was then plotted using absorbance values as the ordinate and the culture time as the abscissa.

### 2.5 Luciferase assay

In order to investigate the regulatory relationship of HOTAIR/miR‐138, miR‐138/TIMP1 and oestrogen/HOTAIR, the 3’ untranslated regions (3’UTRs) of HOTAIR and TIMP1 were cloned into pcDNA vectors (Promega). Subsequently, the locations of miR‐138 binding sites on the 3’UTRs of HOTAIR and TIMP1 were identified using a biological prediction website, microRNA.org. In the next step, site‐directed mutagenesis was carried out in the miR‐138 binding sites to generate mutant 3’UTRs of HOTAIR and TIMP1, which were subsequently cloned into pcDNA vectors. Finally, the effect of miR‐138 on the expression of HOTAIR and TIMP1 was assayed by transfecting HFOB and MG63 cells with both wild‐type and mutant plasmids. Furthermore, transfected HFOB and MG63 cells were treated with 5 nmol/L and 10 nmol/L of oestrogen, respectively, for 24 hours before the luciferase activity in the cells was measured to evaluate the effect of oestrogen on the transcription of HOTAIR promoter. In particular, the luciferase activity of transfected cells was measured on a Glomax20/20 luminometer (Promega) using a Dual‐luciferase assay kit (Promega). Each experiment was repeated three times.

### 2.6 Western blot analysis

After the tissue and cell samples were collected, they were lysed using a cell lysis buffer before the protein concentration in the lysate was measured by a BCA kit (Thermo Fisher Scientific). Subsequently, 50 μg of proteins were taken from each sample, resolved by 10% sodium dodecyl sulphate‐polyacrylamide gel (SDS‐PAGE) electrophoresis, transferred onto a polyvinylidene fluoride (PVDF) membrane, blocked at 37°C for 1 hour using 5% skimmed milk, and incubated with anti‐TIMP1 (1:1000, Millipore) and anti‐β‐actin primary antibodies (internal control, 1:1000, Santa Cruz Biotechnology) overnight at 4°C. After the membrane was washed by PBST, it was further incubated at room temperature for 2 hours with horseradish peroxidase (HRP) labelled secondary antibodies (1:2000, Santa Cruz Biotechnology). Subsequently, the membrane was developed using an ECL solution (Amersham Bioscience) and visualized using a Scion Image analysis system (Scion Corporation). The relative expression of TIMP1 protein was calculated based on the ratio between the optical density of TIMP1 protein and that of the internal control β‐actin.

### 2.7 Apoptosis analysis

After 48 hours of transfection, transfected cells were collected and stained using an Annexin‐V‐Fluorescein isothiocyanate (Annexin‐V‐FITC) kit for cell apoptosis detection (Sigma‐Aldrich). The apoptosis of the cells was detected by flow cytometry using an excitation wavelength of 488 nm and an emission wavelength of 525 nm (for the detection of PI) or an excitation wavelength of 488 nm and an emission wavelength of 578 nm (for the detection of Annexin‐V) (BD FACSCANTO II).

### 2.8 Immunohistochemistry (IHC) assay

The samples of formalin‐fixed demineralized bone tissues and treated cells were fixed in a 10% formaldehyde solution, soaked in paraffin, dewaxed with xylene, hydrated with gradient alcohol, treated for 10 minutes with PBS containing 0.2% Triton X‐100, blocked for 30 minutes with PBS containing 3% goat serum and then incubated overnight at 4°C with anti‐TIMP1 primary antibodies. On the next day, the samples were rinsed with PBS and incubated for 30 minutes with biotin‐labelled secondary antibodies (Abcam). We used peroxidase 3,3‐diaminobenzidine (DAB) to perform the staining and then used haematoxylin to perform the counterstaining. Subsequently, the samples were observed under a microscope to identify the cells showing positive expression of TIMP1.

### 2.9 Statistical analysis

SPSS 20.0 software was used for data analysis. Measurement data were presented as mean ± standard deviations. Student's *t* tests were used for the comparison between two groups, while one‐way analysis of variance (ANOVA) was used for the comparison among multiple groups, followed by Bonferroni's test. All tests were two‐sided, with *P* < .05 considered as statistically significant.

## 3 RESULTS

### 3.1 HOTAIR was down‐regulated in post‐menopausal females

**FIGURE 1:**
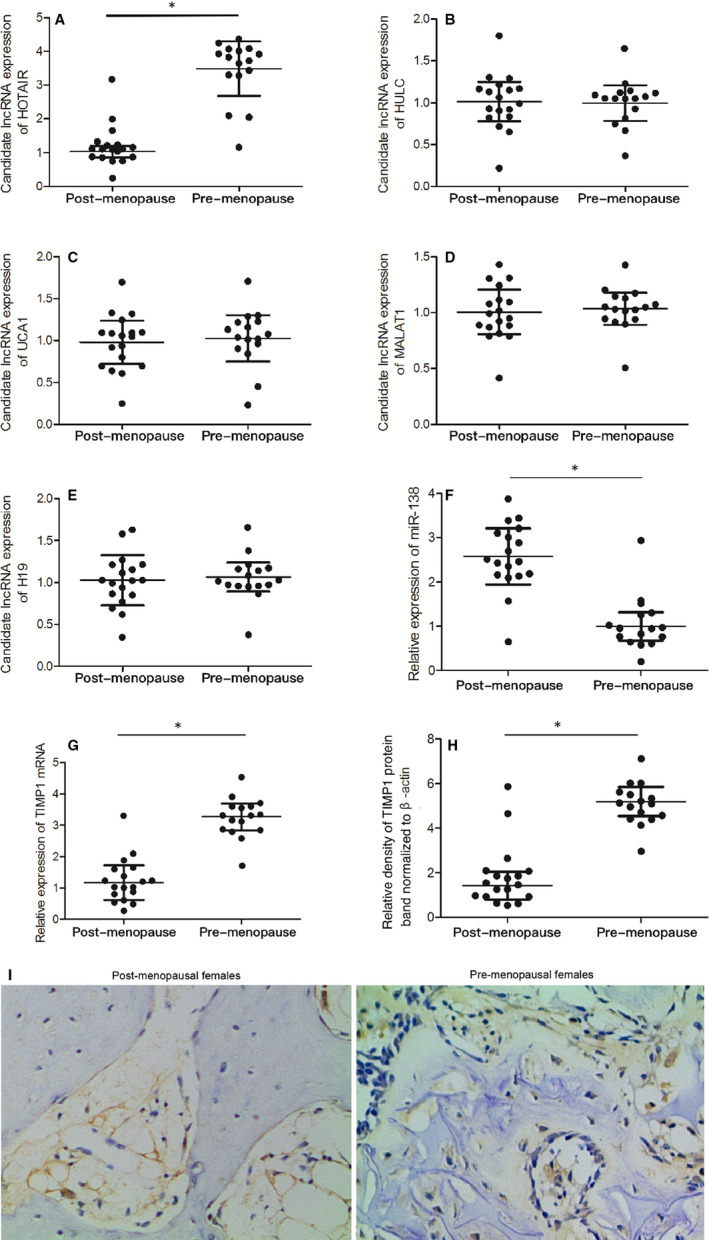
The expression of HOTAIR and TIMP1 was reduced while the expression of miR‐138 was elevated in post‐menopausal females compared with that in pre‐menopausal females (Replicate number N = 3). A, Expression levels of HOTAIR were significantly down‐regulated in post‐menopausal females compared with pre‐menopausal females (*P value of < .05 compared with the pre‐menopausal females). B, Expression levels of HULC were similar between pre‐menopausal and post‐menopausal females. C, Expression levels of UCA1 were similar between pre‐menopausal and post‐menopausal females. D, Expression levels of MALAT1 were similar between pre‐menopausal and post‐menopausal females. E, Expression levels of H19 were similar between pre‐menopausal and post‐menopausal females. F, Relative expression of miR‐138 was elevated in post‐menopausal females compared with pre‐menopausal females (*P value of < .05 compared with the pre‐menopausal females). G, Relative expression of TIMP1 mRNA was significantly reduced in post‐menopausal females compared with pre‐menopausal females (*P value of < .05 compared with the pre‐menopausal females). H, The TIMP1 protein band was less visible in post‐menopausal females compared with pre‐menopausal females (*P value of < .05 compared with the pre‐menopausal females). I, IHC assays showed a higher level of TIMP1 in the pre‐menopausal group compared with that in the post‐menopausal group

In this study, bone tissue samples were collected from 34 osteoporosis patients, including 18 post‐menopausal females and 16 pre‐menopausal females. The demographic parameters, risk factors and BMD of the subjects were presented in Table 1. To identify the potential lncRNAs implicated in the development of osteoporosis in post‐menopausal women, we compared the expression of several lncRNAs between the post‐menopausal group and the pre‐menopausal group. As shown in Figure 1, the expression of HOTAIR (Figure 1A) in post‐menopausal females was significantly down‐regulated. Meanwhile, the expression levels of HULC (Figure 1B), UCA1 (Figure 1C), MALAT1 (Figure 1D) and H19 (Figure 1E) were similar between pre‐menopausal and post‐menopausal females.

### 3.2 The negative correlation between the expression of miR‐138 and TIMP1/HOTAIR

Subsequently, the expression of miR‐138 and TIMP1/HOTAIR was compared between the post‐menopausal and pre‐menopausal groups. As shown in Figure 1F, the relative expression of miR‐138 was significantly elevated in the post‐menopausal group compared with that in the pre‐menopausal group. However, the relative expression of both TIMP1/HOTAIR mRNA (Figure 1G) and protein (Figure 1H) was significantly reduced in the post‐menopausal group. The above results were also confirmed by the results of IHC assays (Figure 1I), indicating the presence of a negative correlation between the expression of miR‐138 and TIMP1/HOTAIR.

### 3.3 MiR‐138 could bind to HOTAIR

**FIGURE 2:**
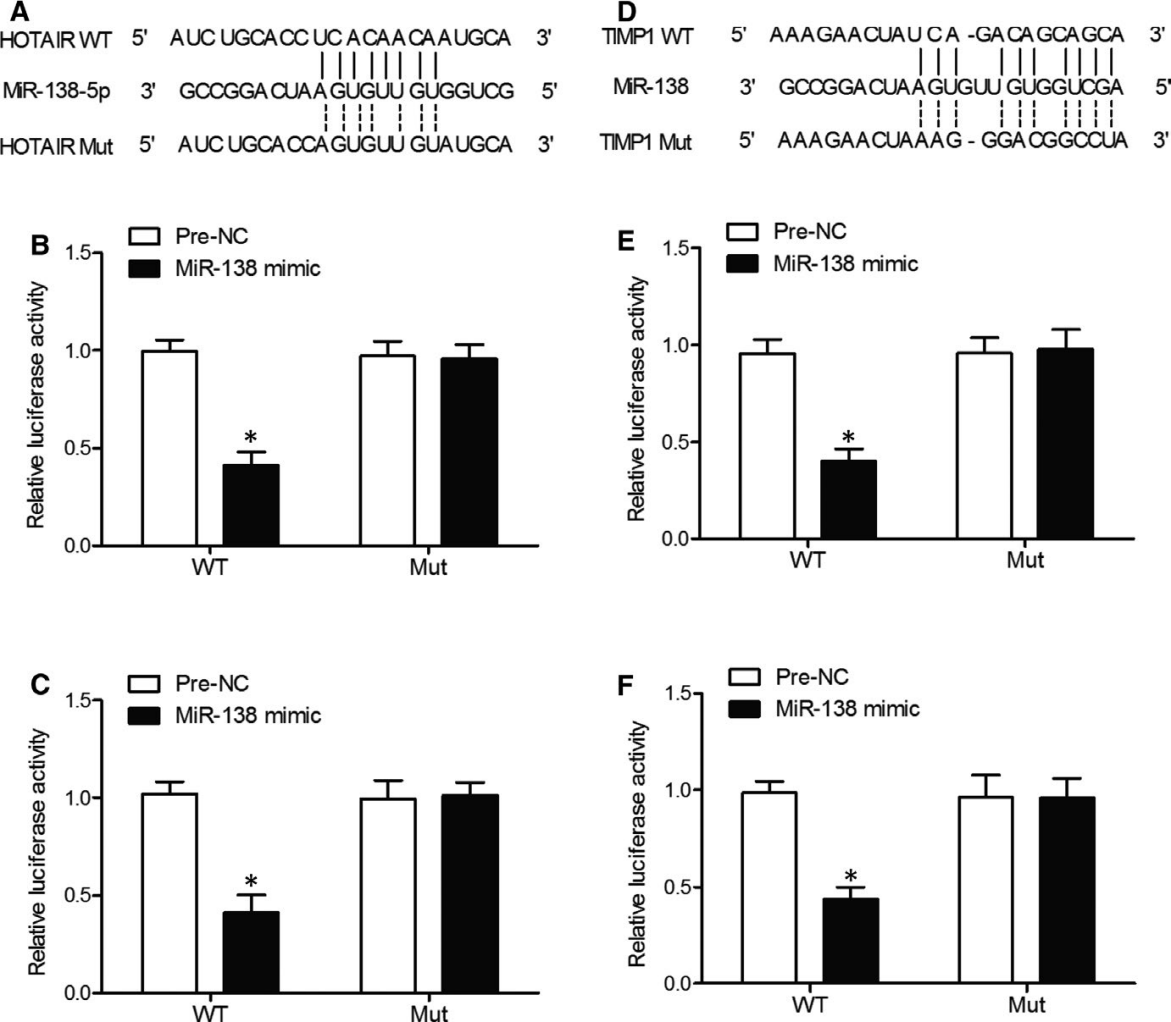
MiR‐138 could bind to HOTAIR while TIMP1 mRNA was a target gene of miR‐138 (Replicate number N = 3). A, A potential ‘seed sequence’ responsible for the binding of miR‐138‐5p to HOTAIR. B, Relative luciferase activity of HFOB cells cotransfected with wild‐type HOTAIR and miR‐138 mimics was evidently higher than other HFOB cell groups (*P value of < .05 compared with the negative controls). C, Relative luciferase activity of MG63 cells cotransfected with wild‐type HOTAIR and miR‐138 mimics was evidently higher than other MG63 cell groups (*P value of < .05 compared with the negative controls). D, A potential ‘seed sequence’ of miR‐138 was located in the 3’UTR of TIMP1. E, Relative luciferase activity of HFOB cells cotransfected with wild‐type TIMP1 mRNA and miR‐138 mimics was evidently higher than other HFOB cell groups (*P value of < .05 compared with the negative controls). F, Relative luciferase activity of MG63 cells cotransfected with wild‐type TIMP1 mRNA and miR‐138 mimics was evidently higher than other MG63 cell groups (*P value of < .05 compared with the negative controls)

To explore the regulatory relationship between miR‐138 and HOTAIR, a computational analysis was conducted using an online miRNA database. As shown in Figure 2A, there was a matching ‘seed sequence’ on miR‐138‐5p allowing it to bind to HOTAIR. To verify the role of this ‘seed sequence’, we cotransfected cells with miR‐138‐5p mimics and wild‐type or mutant HOTAIR, and then measured the relative luciferase activity in transfected cells. As shown in Figure 2B, the relative luciferase activity of HFOB cells cotransfected with wild‐type HOTAIR and miR‐138 mimics was significantly reduced compared with that in the negative control cells, while the relative luciferase activity of HFOB cells cotransfected with mutant HOTAIR and miR‐138 mimics showed no major change. Similar results were also obtained in MG63 cells (Figure 2C), confirming the fact that HOTAIR is a direct target of miR‐138.

### 3.4 TIMP1 mRNA was a target gene of miR‐138

Furthermore, to explore the regulatory relationship between miR‐138 and TIMP1, we carried out a computational analysis using the online target gene predicting tool. As shown in Figure 2D, TIMP1 was identified as a potential target gene of miR‐138 with a miR‐138 binding site located in the 3’UTR of TIMP1. Subsequently, we cotransfected HFOB and MG63 cells with miR‐138 mimics and wild‐type or mutant 3’UTR of TIMP1, and then measured the relative luciferase activity in transfected cells. As shown in Figure 2E, the HFOB cells cotransfected with miR‐138 mimics and the 3’UTR of wild‐type TIMP1 showed apparently decreased luciferase activity compared with the cells transfected with negative controls, while the HFOB cells cotransfected with miR‐138 mimics and the 3’UTR of mutant TIMP1 showed no change in luciferase activity. Similar results were also obtained in MG63 cells (Figure 2F), confirming the fact that TIMP1 acts as a virtual target gene of miR‐138.

### 3.5 Oestrogen exhibited its effect via regulating the level of HOTAIR

**FIGURE 3:**
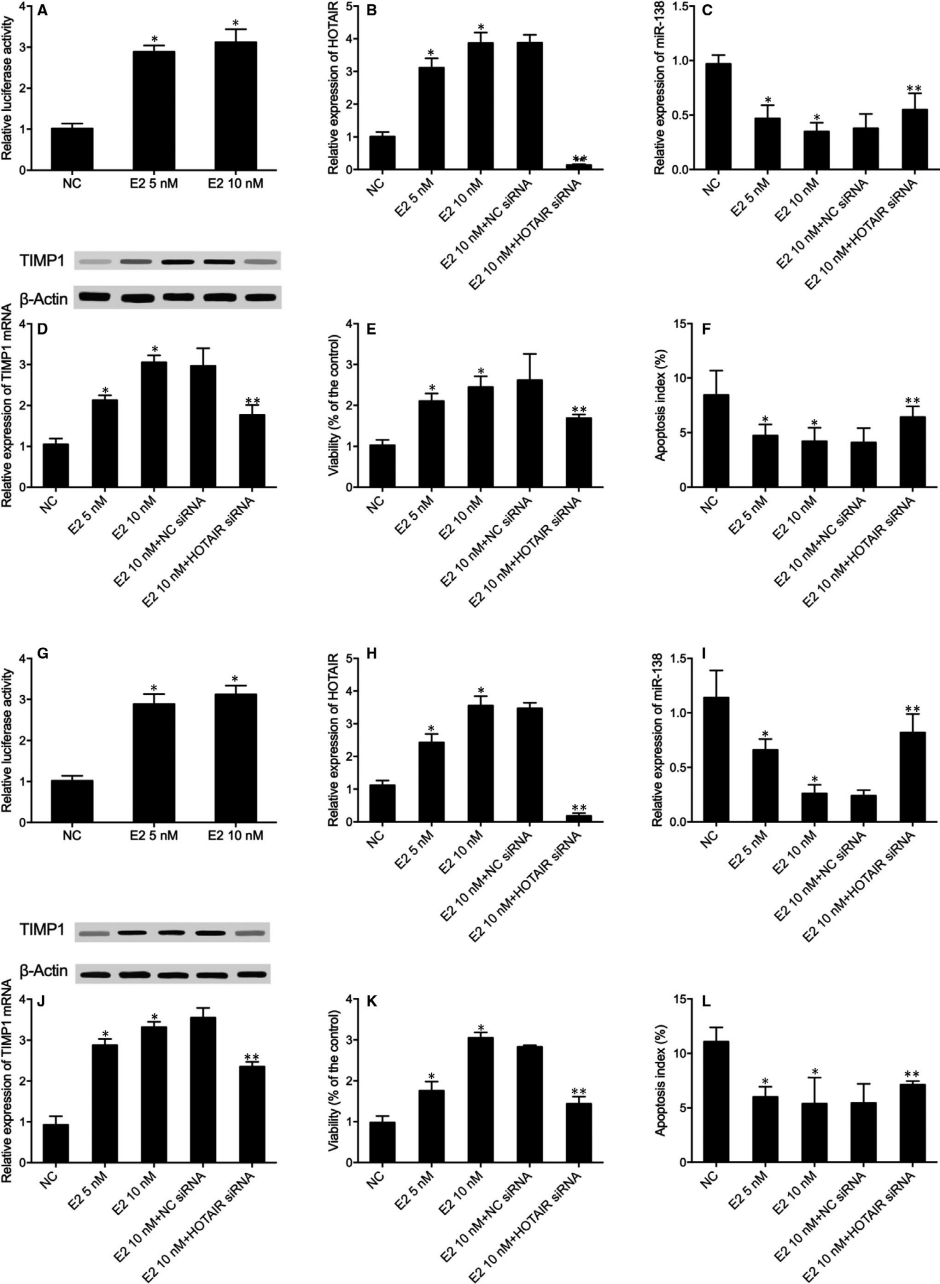
Oestrogen could increase the luciferase activity of HOTAIR while influencing the relative expression of HOTAIR and its downstream targets, miR‐138 and TIMP1. The silencing of HOTAIR could also block the effect of oestrogen on HOTAIR and its downstream targets. Oestrogen could also increase cell viability and suppress cell apoptosis via mediating the HOTAIR/ mir‐138/ TIMP‐1 signalling axis (Replicate number N = 3). A, Relative luciferase activity of HOTAIR was elevated in HFOB cells treated with 5 nmol/L and 10 nmol/L of oestrogen (*P value of < .05 compared with the negative controls). B, Relative expression of HOTAIR was increased in HFOB cells treated with 5 nmol/L and 10 nmol/L of oestrogen, and the transfection of HOTAIR siRNA silenced the expression of HOTAIR (*P value of < .05 compared with the negative controls; ** *P* value of < .05 compared with the E2 10 nmol/L + NC siRNA group). C, Relative expression of miR‐138 was inhibited in HFOB cells treated with 5 nmol/L and 10 nmol/L of oestrogen, and HOTAIR siRNA increased the miR‐138 expression (*P value of < .05 compared with the negative controls; **P value of < .05 compared with the E2 10 nmol/L + NC siRNA group). D, TIMP1 mRNA expression was up‐regulated by 5 nmol/L and 10 nmol/L of oestrogen, and HOTAIR siRNA partly blocked the effect of oestrogen in HFOB cells (*P value of < .05 compared with the negative controls; **P value of < .05 compared with the E2 10 nmol/L + NC siRNA group). E, The viability (% of control) of oestrogen‐treated HFOB cells was dose‐dependently increased, while HOTAIR siRNA reduced cell viability (*P value of < .05 compared with the negative controls; ** *P* value of < .05 compared with the E2 10 nmol/L + NC siRNA group). F, Apoptosis index of HFOB cells was dose‐dependently reduced by oestrogen (5 nmol/L and 10 nmol/L), while silencing of HOTAIR blocked the effect of oestrogen (*P value of < .05 compared with the negative controls; ** *P* value of < .05 compared with the E2 10 nmol/L + NC siRNA group). G, Relative luciferase activity of HOTAIR was elevated in MG63 cells treated with 5 nmol/L and 10 nmol/L of oestrogen (*P value of < .05 compared with the negative controls). H, Relative expression of HOTAIR was increased in HFOB cells treated with 5 nmol/L and 10 nmol/L of oestrogen, and the transfection of HOTAIR siRNA silenced the expression of HOTAIR (*P value of < .05 compared with the negative controls; ** *P* value of < .05 compared with the E2 10 nmol/L + NC siRNA group). I, Relative expression of miR‐138 was inhibited in HFOB cells treated with 5 nmol/L and 10 nmol/L of oestrogen, and HOTAIR siRNA increased miR‐138 expression (*P value of < .05 compared with the negative controls; ** *P* value of < .05 compared with the E2 10 nmol/L + NC siRNA group). J, TIMP1 mRNA and protein expression was up‐regulated by 5 nmol/L and 10 nmol/L of oestrogen, and HOTAIR siRNA partly blocked the effect of oestrogen in HFOB cells (*P value of < .05 compared with the negative controls; ** *P* value of < .05 compared with the E2 10 nmol/L + NC siRNA group). K, The viability (% of control) of oestrogen‐treated HFOB cells was dose‐dependently increased, while HOTAIR siRNA reduced cell viability (*P value of < .05 compared with the negative controls; ** *P* value of < .05 compared with the E2 10 nmol/L + NC siRNA group). L, Apoptosis index of HFOB cells was dose‐dependently inhibited by 5 nmol/L and 10 nmol/L of oestrogen, while HOTAIR silencing promoted cell apoptosis (*P value of < .05 compared with the negative controls; ** *P* value of < .05 compared with the E2 10 nmol/L + NC siRNA group)

According to the above results, HOTAIR acted as an upstream regulator in the molecular pathway of HOTAIR, miR‐138 and TIMP1. Since HOTAIR is differentially expressed between post‐menopausal and pre‐menopausal females, the effect of oestrogen on the expression of HOTAIR was also evaluated in this study by treating HFOB and MG63 cells with 5 nmol/L or 10 nmol/L of oestrogen, respectively. As shown in Figure 3A, the relative luciferase activity of HFOB cells was the lowest in the negative control group and the highest in the 10 nmol/L oestrogen treatment group, indicating that oestrogen acted as an enhancer of HOTAIR expression.

The relative expression of HOTAIR and its downstream targets, miR‐138 and TIMP1, was also measured in cells treated with 5 nmol/L or 10 nmol/L of oestrogen, respectively. Accordingly, the relative expression of HOTAIR (Figure 3B), as well as the relative expression of TIMP1 mRNA and protein (Figure 3D), in the oestrogen‐treated HFOB cells was gradually increased in a dose‐dependent manner. On the contrary, the relative expression of miR‐138 (Figure 3C) gradually decreased over an increasing dose of oestrogen. It is noteworthy that the silencing of HOTAIR by HOTAIR siRNA (Figure 3B) blocked the regulatory effect of oestrogen on HFOB cells treated with 10 nmol/L of oestrogen.

Moreover, a gradual increase of cell viability (% of control, Figure 3E) and a stepwise decrease in cell apoptosis index (Figure 3F) were observed along with the administration of 5 nmol/L or 10 nmol/L of oestrogen in HFOB cells, while the transfection of HOTAIR siRNA partly restored the oestrogen‐induced up‐regulation of cell viability and down‐regulation of cell apoptosis. Similar results were obtained when repeating the above observations in MG63 cells (Figure 3G‐L), validating that oestrogen could exhibit a therapeutic effect on the osteoporosis of post‐menopausal females via mediating the HOTAIR/ mir‐138/ TIMP‐1 signalling axis.

## 4 DISCUSSION

As a disease of bone degeneration frequently observed at an older age, osteoporosis is characterized by changes in bone density and bone micro‐structures.^28^ During osteoporosis, the dynamic equilibrium between bone formation and bone absorption is impaired.^29^ During bone formation, osteoblasts play an important role in bone growth, bone metabolism and bone repair.^30^ Therefore, the dysfunction in osteoblast differentiation, proliferation and growth can induce the onset of osteoporosis. From this point of view, the apoptosis of osteoblasts can be considered as an important step in the pathogenesis of osteoporosis.^31^ Since post‐menopausal women are predisposed to the apoptosis of osteoblasts due to their decreased level of oestrogen, they also tend to suffer from decreased bone mineral density (BMD) and subsequent fractures. Also, osteoporosis‐induced fractures are currently considered by the World Health Organization as a major public health issue in post‐menopausal women. Therefore, the prevention of osteoporosis is essential among post‐menopausal women.^32^

Interestingly, the mechanisms underlying the role of MALAT1 and HOTAIR in impaired hormone responses are substantially different. When the level of HOTAIR in the body is decreased, the transcription of the target genes of HOTAIR is elevated above their basal levels and the oestrogen responsiveness is blunted due to the increase in the basal transcription of target genes. On the other hand, the knockdown of MALAT1 results in blocked oestrogen responses due to the elevated basal transcription of the target genes of MALAT1.^25^ In this study, we compared the expression of several lncRNAs between post‐menopausal and pre‐menopausal females. Unlike that of HULC, UCA1, MALAT1 and H19, the expression of HOTAIR was apparently decreased in the post‐menopausal group compared with that in the pre‐menopausal group. In addition, we conducted a computational analysis to identify the location of the miR‐138‐5p binding site in HOTAIR. Subsequently, the relative luciferase activity of cells cotransfected with wild‐type HOTAIR and miR‐138 mimics was obviously decreased, suggesting that HOTAIR acted as a target of miR‐138.

HOTAIR is a lncRNA implicated in the pathogenesis of several diseases, including cardiovascular diseases and cancers.^33^, ^34^, ^35^ A recent study showed that HOTAIR may be used as a diagnostic biomarker for the treatment of RA.^36^ Nevertheless, the mechanisms underlying the roles of HOTAIR in the development of RA still require further investigation. For example, miR‐138 has been shown to participate in the phenotype selection of chondrocytes, indicating that miR‐138 may be associated with RA development. In another study, the role of HOTAIR in the proliferation of chondrocytes and LPS‐induced inflammation has been investigated during the pathogenesis of RA.^14^ In this study, TIMP1 was identified via the computational analysis as a potential target gene of miR‐138. When compared with that in the cells transfected with negative controls, the relative luciferase activity of cells cotransfected with wild‐type TIMP1 and miR‐138 mimics was significantly reduced, indicating that TIMP1 acted as a target of miR‐138.

TIMP1 has been implicated in the metabolism of bones. In addition, the increased expression of TIMP1 in osteoblasts has been shown to increase mineral density and bone mass.^37^ Xie et al also demonstrated that the inhibition of TIMP1 expression could lead to the apoptosis of osteoblasts.^38^ Furthermore, TIMP1 was found to reduce the number of neuronal cells in patients with neurodegenerative diseases who were exposed to ER stress.^39^ On the other hand, miR‐138 could apparently suppress the differentiation of human stromal stem cells into osteoblasts.^40^, ^41^ Furthermore, miR‐138 was also shown to inhibit the differentiation and proliferation of skeletal cells by inducing their apoptosis.^41^ Therefore, miR‐138 may be used as a novel target in the treatment of osteoporosis. Nevertheless, the function of miR‐138 in the development of osteoporosis requires further investigation.^27^ In this study, we also measured the expression of miR‐138 and TIMP1 in bone tissue samples collected from osteoporosis patients. Compared with that in the pre‐menopausal group, miR‐138 expression in the post‐menopausal group was much higher. Meanwhile, the mRNA and protein expression of TIMP1 was lower in the post‐menopausal group compared with that in the pre‐menopausal group. The above results collectively indicated the presence of a negative correlation between the expression of miR‐138 and TIMP1/HOTAIR. Meanwhile, the effect of oestrogen on HOTAIR expression was evaluated by measuring the luciferase activity of HOTAIR in cells treated with 5 nmol/L or 10 nmol/L of oestrogen, respectively. A dose‐dependent increase in the luciferase activity was observed after oestrogen treatment, indicating that oestrogen could elevate the expression of HOTAIR. Furthermore, the increased expression of miR‐138 reduced the expression of both HOTAIR and TIMP1. Finally, a higher expression level of TIMP1 protein also increased the viability and reduced the apoptosis of osteoblasts, confirming the role of HOTAIR in the pathogenesis of osteoporosis among post‐menopausal females. Xie et al have shown that the expression of TIMP1 could prevent the apoptosis of osteoblasts via the suppression of MMP activity.^38^ However, the study of Xie et al failed to clarify the mechanisms underlying the role of TIMP1 in promoting the apoptosis of osteoblasts. Therefore, although this study suggested a novel role of TIMP1 in the acceleration of osteoblast apoptosis, it remains unclear whether ER stress and miR‐138 are significantly involved in osteoporosis. Nevertheless, another study has shown that ER stress and miR‐138 expression could both activate the apoptotic pathway in osteoblasts,^27^ suggesting that these two factors indeed cross‐talk with each other during the pathogenesis of osteoporosis.

## 5 CONCLUSION

In conclusion, our findings demonstrated a novel role of oestrogen in the development of osteoporosis by regulating the expression of HOTAIR. In addition, HOTAIR acted as a competing endogenous RNA for miR‐138 and its target gene TIMP1, which in turn plays an important role in the apoptosis of osteoblasts. Finally, we confirmed that oestrogen could at least partially affect the progress of osteoporosis by controlling the apoptosis of osteoblasts and by up‐regulating the expression of HOTAIR, an lncRNA playing a protective role against osteoporosis.

## CONFLICT OF INTEREST

The authors declare that they have no conflicts of interest.

## AUTHOR CONTRIBUTION

**Shao‐yong Xu:** Conceptualization (equal); Investigation (equal); Methodology (equal); Project administration (equal); Resources (equal); Validation (equal); Writing‐original draft (equal). **Peng Shi:** Investigation (equal); Methodology (equal); Software (equal); Visualization (equal). **Rui‐ming Zhou:** Investigation (supporting); Project administration (lead); Supervision (lead); Writing‐review & editing (lead).

## Data Availability

The data that support the findings of this study are available from the corresponding author upon reasonable request.
